# Efficacy and safety of olanzapine for treatment of patients with bipolar depression: Japanese subpopulation analysis of a randomized, double-blind, placebo-controlled study

**DOI:** 10.1186/1471-244X-13-138

**Published:** 2013-05-14

**Authors:** Hideaki Katagiri, Mauricio Tohen, David P McDonnell, Shinji Fujikoshi, Michael Case, Shigenobu Kanba, Michihiro Takahashi, Juan-Carlos Gomez

**Affiliations:** 1 Lilly Research Laboratories, Sannomiya Plaza Bldg., 7-1-5, Isogamidori, Chuo-ku, Kobe 651-0086, Japan; 2 University of New Mexico Department of Psychiatry, Albuquerque, New Mexico, USA; 3 Lilly Research Laboratories, Eli Lilly and Company, Indianapolis, Indiana, USA; 4 Kyushu University, Fukuoka, Japan; 5 East Asian Bipolar Forum, Fukuoka, JAPAN; 6 Takahashi Psychiatric Clinic, Ashiya, Hyogo, Japan

**Keywords:** Bipolar disorder, Bipolar depression, Olanzapine, Efficacy, Safety, Monotherapy, Japan, Japanese

## Abstract

**Background:**

The efficacy and safety of olanzapine monotherapy are evaluated in Japanese patients from a large, global study of bipolar depression.

**Methods:**

This is an analysis of Japanese patients from a 6-week, global (Japan, China, Korea, Taiwan, and the United States), randomized, double-blind, placebo-controlled, Phase 3 study of patients with a depressive episode of bipolar I disorder. The primary outcome was baseline-to-endpoint change in the Montgomery-Åsberg Depression Rating Scale (MADRS) total score. Secondary outcome measures included the Clinical Global Impressions-Bipolar Version Severity of Illness Scale (CGI-BP), the 17-item Hamilton Depression Rating Scale (HAMD-17) total score, the Young Mania Rating Scale (YMRS) total score, and rates of response (≥50% baseline-to-endpoint reduction in MADRS total score), recovery, and remission.

**Results:**

Of the 156 Japanese patients, 104 had been allocated to olanzapine and 52 to placebo. All results are baseline-to-endpoint change. Compared to placebo, patients in the olanzapine group experienced greater improvement in the primary outcome measure, MADRS total score (−14.9 vs. −10.7; p = .01). They also had greater reductions in the following secondary measures: CGI- BP Depression (−1.41 vs. -0.89; p = .008), CGI-BP Bipolar (−1.31 vs. −0.83; p = .01), HAMD-17 (−11.7 vs. −7.9; p < .01), and YMRS (-0.32 vs. 0.34; p = .03). Differences in rates of response, recovery, and remission were not statistically significant. A greater proportion of olanzapine-treated patients reported treatment- emergent adverse events (87.5% vs. 59.6%; p < .001). Patients treated with olanzapine had greater increases in weight (p < .001) and fasting total cholesterol (p = .008); fasting triglycerides (p = .02), and fasting low-density lipoprotein (p = .01). There was a greater reduction in fasting high-density lipoprotein in olanzapine-treated patients (p = .01). Compared with placebo-group patients, more olanzapine-group patients shifted from borderline to high cholesterol (25.0% vs. 0.0%; p = .007) and had clinically significant weight gain (≥7% body weight) (20.2% vs. 1.9%; p = .001).

**Conclusions:**

Results of this analysis support the efficacy and tolerability of olanzapine for the treatment of bipolar depression in Japanese patients. Results in this population were consistent with those seen in the more ethnically diverse parent study. In making treatment decisions for individual patients, clinicians should carefully consider the risks and benefits of olanzapine treatment.

**Trial Registration:**

Clinicatrials.gov ID NCT00510146 Olanzapine Treatment of Patients with Bipolar I Disorder

## Background

Bipolar disorder is a devastating condition with a global lifetime prevalence of 2.4% [1] that is characterized by episodic alterations in energy level, cognition, and mood consistent with mania, depression, or mixed states of manic and depressive symptoms. Multiple agents have shown efficacy in the treatment of bipolar mania including lithium, valproate, and all currently available atypical antipsychotics [2-4]. When managing bipolar depression, a tricyclic antidepressant, norepinephrine-dopaminergic reuptake inhibitor, or selective serotonin reuptake inhibitor is frequently combined with a mood-stabilizing agent [5,6], though the efficacy and safety of this strategy is not well-established.

More recently, atypical antipsychotics have been used as monotherapy for bipolar depression. Efficacy was demonstrated in 2 randomized, placebo-controlled trials of quetiapine for treatment of depressive episodes in patients with bipolar I and bipolar II disorder [7,8]. In contrast, efficacy was not shown in randomized, placebo-controlled studies of aripiprazole [9-11] and ziprasidone [12] as monotherapy for acute bipolar depression.

In a single 8-week, double-blind protocol comparing olanzapine, olanzapine-fluoxetine, and placebo which was conducted as 2 contemporaneous, identical studies, both treatments were more effective than placebo when study results were combined [13]. When analyzed separately, however, a significant advantage over placebo was seen with combination therapy in both studies, while olanzapine as monotherapy was more effective than placebo in only 1 of the 2 studies (Data on File, Eli Lilly and Company).

To further evaluate the effectiveness of olanzapine monotherapy for the treatment of depressive episodes in bipolar I disorder, a large, international, double-blind, placebo-controlled trial was undertaken [14]. After 6 weeks of treatment, patients allocated to olanzapine had significantly greater baseline-to-endpoint improvement in the Montgomery-Åsberg Depression Rating Scale (MADRS) [15] total score compared with patients allocated to placebo (−13.8 vs. −11.7; p = .02). Likewise, with response defined a priori as a ≥50% reduction in MADRS total score at endpoint, the olanzapine group had a significantly greater response than the placebo group (52.5% vs. 43.3%, p = .0498).

Prior to 2012, there were no medication options with well-established evidence approved in Japan for treatment of bipolar depression. Though compelling data existed for treatment of bipolar depression in Western populations, it was unclear whether these findings would hold true for a cohort of Japanese patients. The olanzapine monotherapy trial described above included a large cohort of Japanese patients and, to our knowledge, was the first double-blind, placebo-controlled trial to do so. Based on the results of this trial, Japan became the first country in the world to approve olanzapine monotherapy for treatment of bipolar depression. Though olanzapine monotherapy has been used for the treatment of this condition in Japan, limited data have been published. Therefore, we analyzed efficacy and safety data from Japanese patients who were enrolled in the larger study described above, so that clinicians who care for Japanese patients will have a more comprehensive understanding of its treatment profile in this population.

## Methods

### Patients

Patients from the parent study were men and women inpatients and outpatients, age 18 to 64 years. They were recruited in Japan, China, Taiwan, Korea, and the United States and met diagnostic criteria for a major depressive episode and for bipolar I disorder (Diagnostic and Statistical Manual of Mental Disorders, fourth edition, text revision). Only patients who were recruited in Japan were included in this subanalysis. At the time of randomization, all patients were in a depressive episode that had lasted 90 days or less, and was defined by a 17-item Hamilton Depression Rating Scale (HAMD-17) [16] total score ≥18. None were actively in a manic episode, defined as having a Young Mania Rating Scale (YMRS) [17] total score ≤8. All patients had a history of at least 1 manic or mixed episode in the previous 6 years. Exclusion criteria included unstable medical illness; history of diabetes, hemoglobin A_1c_ ≥6.5% or blood glucose level indicative of diabetes; history of serious psychiatric illness other than bipolar depression; current rapid cycling mood disturbance; recent use of clozapine, depot antipsychotics, or central nervous system medications other than mood stabilizers; and recent history of substance dependence.

### Study design

This was a 6-week, randomized, double-blind, placebo-controlled, Phase 3 study of olanzapine as monotherapy for treatment of patients with bipolar I disorder who were acutely depressed. The study was approved by the relevant institutional ethics committee at each center and was conducted in compliance with the Declaration of Helsinki. Names of the specific review boards can be found in the Ethics Approval section at the end of this manuscript. Following a complete description of the study and prior to initiation of any study drug or procedure, written informed consent was obtained from the patient. Patients who met all inclusion criteria and no exclusion criteria were then randomly assigned in a 2:1 ratio to olanzapine (5-20 mg/day at the discretion of the investigator) or placebo. Patients were evaluated at least weekly through Week 6. Data collected beyond the acute phase portion of the study were not included in this analysis.

### Assessment measures

Efficacy was assessed using change from baseline to endpoint in total scores of the following assessment tools:

•MADRS: A clinician-administered interview regarding symptoms of depression in the previous week, and consisting of 10 items, each scored from 0 to 6 in increasing order of severity.

•Clinical Global Impressions-Bipolar Version Severity of Illness Scale (CGI-BP) [18]: A clinician rating of global symptom severity at the time of assessment relative to other patients with bipolar depression. Symptom severity is scored from 1 to 7, where 1 equals normal and 7 equals extremely ill.

•HAMD-17: A 17-item, multiple-choice questionnaire in which based on interview and observation, the clinician must choose the best possible response regarding a patient’s severity of depression. Each question has between 3 and 5 possible responses, and total scores range from 0 to 52, where higher values indicate greater severity.

The potential to induce manic symptoms was assessed using the following tool:

•YMRS: An 11-item, multiple-choice questionnaire regarding symptoms of mania. Rated by the clinician, the YMRS total score ranges from 0 to 60, where higher scores indicate greater severity.

Rates of response, remission, and recovery were assessed. Response was defined as a baseline-to-endpoint reduction of ≥50% in MADRS total score, and recovery was defined as a score ≤12 in the MADRS total score for at least 4 weeks and completion of the 6-week study. Remission was a priori defined as a MADRS total score ≤12 (called partial remission in this manuscript), and was defined post hoc as a MADRS total score ≤8 (as recommended by the International Society of Bipolar Disorder [ISBD], called full remission in this manuscript) [19]. Additionally, study discontinuation and emergence of mania were assessed.

### Safety evaluation

All adverse events occurring during the course of the study were documented and vital signs and weight were assessed at each visit. Electrocardiograms and laboratory analytes, including measures of fasting glucose and lipids, were assessed at baseline and endpoint. Patients were screened for the emergence of extrapyramidal symptoms at every visit using the Drug-induced Extrapyramidal Symptoms Scale [20] (DIEPSS). At every visit, patients were also assessed for suicidality using the Mini International Neuropsychiatric Interview [21] (MINI) and for the emergence of mania (YMRS total score <8).

### Statistical analysis

Analyses were based on the intention-to-treat set of Japanese patients. All tests of treatment effect were conducted at a 2-sided significance level of 0.05, and no adjustments were made for multiple comparisons. Continuous data were assessed using analysis of covariance models with type III sums of squares with a term for treatment and with the baseline measurement value included as a covariate. For continuous data where a baseline measurement was not applicable, analysis of variance (ANOVA) models were used, with a term for treatment. The primary analysis of change from baseline to endpoint in MADRS total score was based on last-observation-carried-forward (LOCF) change. Post hoc analyses of MADRS and the MADRS-6 subscale, which focuses on the “core” symptoms of depression (as assessed by the MADRS items: Apparent Sadness, Reported Sadness, Inner Tension, Lassitude, Inability to Feel, and Pessimistic Thoughts) [22,23] were preformed. As a sensitivity analysis, observed-case ANOVA and mixed-effects model repeated measures (MMRM) were used to assess changes in MADRS total score. For analyses of proportions, the Fisher exact test was used.

## Results

### Patient disposition

**Figure 1:**
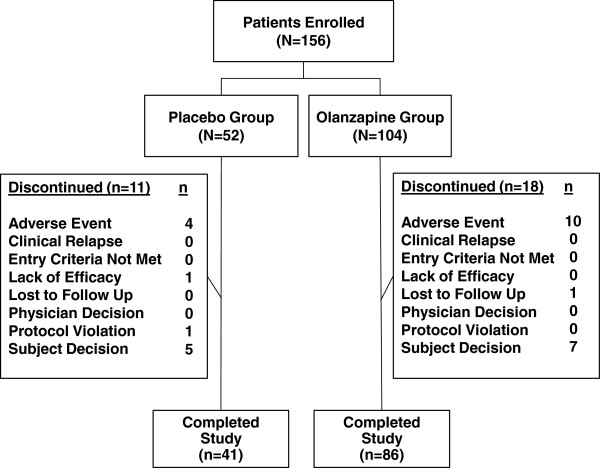
Patient flow diagram.

Of the 514 patients randomly allocated to treatment in the parent study, 156 were Japanese. As shown in Figure 1, the acute phase of the study was completed by 86 of the 104 Japanese patients randomized to olanzapine (82.7%) and 41 of the 52 Japanese patients randomized to placebo (78.8%; p = .66). Patients treated with olanzapine received a mean (standard deviation, [SD]) daily dose of 9.98 (3.14) mg.

At baseline, treatment groups did not differ with regard to gender distribution or the number of prior episodes of mania, depression, or mixed symptoms. Patients in the olanzapine group were significantly older (40.0 [SD 11.0] years vs. 36.3 [SD 9.5] years; p = .04), had been older at the time of illness onset (33.1 [SD 10.9] years vs. 29.1 [SD 8.8] years; p = .02), and weighed less at baseline (59.0 [SD 12.4] kg vs. 64.0 [SD 12.1] kg; p = .02) than those in the placebo group. There were no between-group differences for baseline MADRS total score, HAMD-17 total score, YMRS total score, or any of the CGI-BP subscores (Manic, Depressed, or Bipolar) (Table 1).

**Table 1:** Baseline demographics, illness history, and baseline illness severity

	**Placebo**	**Olanzapine**
	**(N = 52)**	**(N = 104)**
Female gender, n (%)	27 (51.9)	63 (60.6)
Age in years, mean (SD)	36.3 (9.5)	40.0 (11.0)
Age in years at onset of BD, mean (SD)	29.1 (8.8)	33.1 (10.9)
Prior episodes, mean (SD)		
Manic	3.02 (3.07)	3.32 (4.46)
Depressive	3.77 (3.35)	4.54 (5.69)
Mixed	0.23 (0.70)	0.13 (0.48)
Weight in kg, mean (SD)	64.02 (12.14)	59.04 (12.35)
Illness severity scores, mean (SD)		
MADRS total	28.62 (8.01)	29.00 (6.15)
YMRS total	0.85 (1.32)	0.88 (1.32)
HAMD-17 total	22.69 (4.08)	23.10 (3.78)
CGI-BP		
Depression	4.23 (0.73)	4.35 (0.75)
Mania	1.02 (0.14)	1.03 (0.17)
Bipolar	4.10 (0.87)	4.17 (0.86)

Abbreviations: BD = bipolar disorder; CGI-BP = Clinical Global Impressions-Bipolar Version Severity of Illness; HAMD-17 = Hamilton Depression Rating Scale-17 items; MADRS = Montgomery-Åsberg Depression Rating Scale; SD = standard deviation; YMRS = Young Mania Rating Scale.

### Primary efficacy outcome

**Figure 2:**
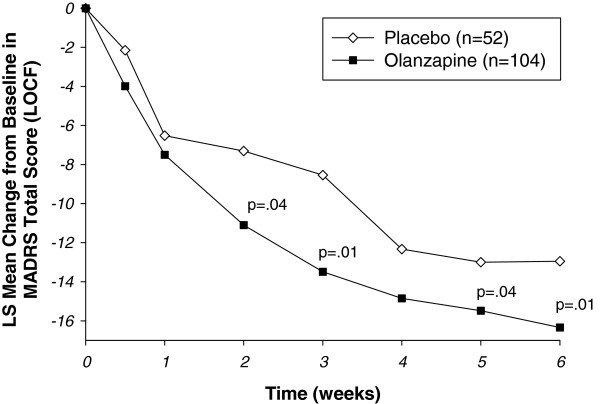
**Visit-wise change from baseline in LS mean MADRS total score (Last observation carried forward methodology).** Abbreviations: MADRS = Montgomery-Åsberg Depression Rating Scale; LS mean = least squares mean.

Baseline-to-endpoint (Week 6) change in MADRS total score was statistically different between groups, with the olanzapine group having greater least-squares (LS) mean score reductions compared with the placebo group (−14.9 [standard error; SE 1.0] vs. −10.7 [SE 1.4]; p = .01). The effect size for the primary outcome was 0.42 (95% confidence interval 0.09, 0.76). Visit-wise changes in LS mean MADRS total score are shown in Figure 2. The olanzapine group had a significantly greater reduction in score than the placebo group, beginning as early as Week 2 (−10.4 [SE 0.8] vs. −7.6 [SE 1.1]; p = .04). Sensitivity analyses using observed case (Week 2: -11.10 vs. -7.31, p = .006; Week 6: -16.34 vs. -12.95, p = .04) and MMRM (Week 2: -11.10 vs. -7.38, p = .006; Week 6: -16.36 vs. -12.91, p = .046) methodologies yielded similar results (Additional file 1: Figures S1 and Additional file 2: Figure S2).

### Secondary efficacy outcomes

As shown in Table 2, efficacy in treating bipolar depression was further supported by significantly greater baseline-to-endpoint improvement in the HAMD-17 total score for the olanzapine group than for the placebo group (−11.7 [SE 0.74] vs. −7.9 [SE 1.04]; p = .004). Olanzapine treatment was superior to placebo in clinician-based assessments of global illness severity. Baseline-to-endpoint improvement was significantly greater for both the CGI-BP Depression scale (−1.4 [SE 0.11] vs. −0.9 [SE 0.16]; p = .008) and the CGI-BP Bipolar scale (−1.3 [SE 0.11] vs. −0.8 [SE 0.15]; p = .01). Patients had a low level of mania symptoms at baseline, and the mean change at endpoint in YMRS total scores improved for the olanzapine group and worsened for the placebo group (−0.32 [SE 0.17] vs. 0.34 [SE 0.24]; p = .03). No patients in the olanzapine group or in the placebo group met criteria for the emergence of mania (Table 2).

**Table 2:** Secondary efficacy measures

**Baseline-to-endpoint change**	**Placebo**	**Olanzapine**	**p Value**
	**(n = 52)**	**(n = 104)**	
	**LS Mean (SE)**	**LS Mean (SE)**	
CGI-BP			
Depression	−0.89 (0.16)	−1.41 (0.11)	.008
Mania	0.03 (0.04)	0.01 (0.03)	.68
Bipolar	−0.83 (0.15)	−1.31 (0.11)	.01
HAMD-17 total	−7.88 (1.04)	−11.66 (0.74)	.004
YMRS total	0.34 (0.24)	−0.32 (0.17)	.03

Abbreviations: CGI-BP = Clinical Global Impressions-Bipolar Version Severity of Illness; HAMD-17 = Hamilton Depression Rating Scale-17 items; LS mean = least squares mean; SE = standard error; YMRS = Young Mania Rating Scale.

#### Scores on the individual MADRS items

Changes in baseline-to-endpoint scores on individual items of the MADRS scale and the MADRS-6 subscale are shown in Table 3. Both LOCF and MMRM analyses are represented. In the comparison between olanzapine and placebo groups, patients in the olanzapine group showed significantly greater improvement in Inner Tension, Reduced Sleep, Reduced Appetite, and Pessimistic Thoughts by LOCF analysis. By MMRM analysis, the olanzapine group showed significantly greater improvement than the placebo group in these 4 variables as well as in Reported Sadness and Inability to Feel. Likewise, the olanzapine group showed significantly greater improvement than the placebo group in MADRS-6 subscale scores by both LOCF and MMRM analyses.

**Table 3:** Baseline-to-endpoint change in itemized LS Mean MADRS scores

				**LOCF analysis**			**MMRM analysis**			
			**Baseline**	**Endpoint**	**p Value**^**a**^		**Endpoint**		**p Value**^**b**^	
**MADRS Item**	**T**_**x**_	**N**	**Mean**	**LS Mean**	**Within T**_**x**_	**Between T**_**x**_	**N**	**LS Mean**	**Within T**_**x**_	**Between T**_**x**_
			**(SD)**	**Change**				**Change**		
				**(SE)**				**(SE)**		
Apparent Sadness	PBO	52	3.12 (1.06)	−1.42 (0.19)	<.001	.36	43	−1.47 (0.19)	<.001	.16
	OLZ	104	3.23 (1.10)	−1.63 (0.13)	<.001		87	−1.80 (0.13)	<.001	
Reported Sadness	PBO	52	3.35 (1.23)	−1.56 (0.20)	<.001	.10	43	−1.60 (0.21)	<.001	.03
	OLZ	104	3.57 (1.06)	−1.95 (0.14)	<.001		87	−2.15 (0.15)	<.001	
Inner Tension	PBO	52	2.71 (1.23)	−0.73 (0.16)	<.001	<.001	43	−0.78 (0.16)	<.001	<.001
	OLZ	104	2.70 (1.09)	−1.43 (0.11)	<.001		87	−1.53 (0.12)	<.001	
Reduced Sleep	PBO	52	3.02 (1.63)	−0.75 (0.22)	<.001	<.001	43	−1.01 (0.22)	<.001	.001
	OLZ	104	2.88 (1.63)	−1.83 (0.15)	<.001		87	−1.91 (0.16)	<.001	
Reduced Appetite	PBO	52	1.79 (1.70)	−0.78 (0.16)	<.001	.003	43	−0.91 (0.16)	<.001	.004
	OLZ	104	1.77 (1.52)	−1.35 (0.11)	<.001		87	−1.51 (0.12)	<.001	
Concentration Difficulties	PBO	52	3.65 (1.23)	−1.30 (0.21)	<.001	.75	43	−1.46 (0.22)	<.001	.78
	OLZ	104	3.67 (1.08)	−1.38 (0.15)	<.001		87	−1.53 (0.16)	<.001	
Lassitude	PBO	52	3.10 (1.09)	−1.22 (0.19)	<.001	.32	43	−1.33 (0.20)	<.001	.37
	OLZ	104	3.23 (1.10)	−1.45 (0.13)	<.001		87	−1.56 (0.14)	<.001	
Inability to Feel	PBO	52	3.29 (1.18)	−1.20 (0.20)	<.001	.06	43	−1.24 (0.22)	<.001	.03
	OLZ	104	3.43 (0.91)	−1.66 (0.14)	<.001		87	−1.82 (0.15)	<.001	
Pessimistic Thoughts	PBO	52	3.08 (1.08)	−1.05 (0.19)	<.001	.03	43	−1.04 (0.20)	<.001	.01
	OLZ	104	3.10 (1.02)	−1.54 (0.13)	<.001		87	−1.66 (0.14)	<.001	
Suicidal Thoughts	PBO	52	1.52 (1.0)	−0.68 (0.13)	<.001	.87	43	−0.80 (0.13)	<.001	.95
	OLZ	104	1.41 (0.82)	−0.71 (0.09)	<.001		87	−0.79 (0.09)	<.001	
MADRS-6^c^ Subscale	PBO	52	18.63 (5.02)	−7.16 (0.94)	<.001	.03	43	−7.25 (0.99)	<.001	.008
	OLZ	104	19.26 (3.88)	−9.67 (0.66)	<.001		87	−10.54 (0.70)	<.001	

Abbreviations: LOCF = last observation carried forward; LS mean = least squares mean; MADRS = Montgomery-Åsberg Depression Rating Scale; MMRM = mixed-effects model repeated measures; OLZ = olanzapine group; PBO = placebo group; SD = standard deviation; SE = standard error.

^a^ within treatment p values are from t-tests on LS Mean change; p values and LS Mean for change from baseline are from analysis of covariance model: change = treatment + baseline + geographic region (Type III sums of squares).

^b^ within treatment p values are from t-tests on LS Mean change; p values are from Type III sums of squares and Kenward-Roger approximation as denominator degrees of freedom; MMRM analysis: change = treatment + baseline + geographic region + visit + treatment * visit.

^c^ MADRS-6 subscale contains 6 individual MADRS items: Apparent Sadness, Reported Sadness, Inner Tension, Lassitude, Inability to Feel, and Pessimistic Thoughts, considered to be “core” depressive symptoms.

#### Response, remission, and recovery

There were no significant differences between treatment groups in rates of response, remission, or recovery. However, rates of symptomatic response were numerically greater for the olanzapine group compared with those for the placebo group (54.8% vs. 40.4%, p = .13; Table 4). When analyzed with the post hoc definition of MADRS total score ≤8, rates of full remission and recovery were numerically higher for olanzapine. Rates of partial remission, when defined as a MADRS total score ≤12 at any point in time, were slightly higher for the placebo group (Table 4).

**Table 4:** Response, remission, recovery, and study discontinuation

	**Placebo**	**Olanzapine**	**p Value**
	**(n = 52)**	**(n = 104)**	
	n (%)	n (%)	
Response	21 (40.4)	57 (54.8)	.13
Partial remission (MADRS ≤12)	29 (55.8)	56 (53.8)	.87
Full remission (MADRS ≤8)	18 (34.6)	45 (43.3)	.39
Recovery	5 (9.6)	15 (14.4)	.46
Study discontinuations			
Total	11 (21.2)	18 (17.3)	.66
Due to adverse events	4 (7.7)	10 (9.6)	.78
Due to lack of efficacy	1 (1.9)	0 (0.0)	.33
Lost to follow-up	0 (0.0)	1 (1.0)	1.00

Abbreviations: MADRS = Montgomery-Åsberg Depression Rating Scale.

### Safety measures

Safety data were consistent with those seen in the parent study and with the known safety profile of olanzapine. There were no deaths, and one serious adverse event, hypokalemic periodic paralysis in a patient in the olanzapine group. The rate of discontinuation due to an adverse event did not differ between groups (9.6% for olanzapine and 7.7% for placebo; p = .78). Compared with the placebo group, the olanzapine group had significantly higher rates of treatment-emergent adverse events (87.5% vs. 59.6%; p < .001), over half of which were possibly related to study drug (64.4%). Treatment-emergent adverse events occurring in ≥5% of olanza-pine-treated patients were somnolence (38.5%), weight increase (28.8%), increased appetite (16.3%), nasopharyngitis (14.4%), constipation and thirst (both 7.7%), malaise (6.7%), and elevations of alanine aminotransferase and aspartate aminotransferase (both 5.8%).

After 6 weeks of treatment, patients in the olanzapine group had significantly greater weight gain than patients in the placebo group (2.12 [SE 0.21] kg vs. −0.36 [SE 0.29] kg; p < .001). Also, clinically significant weight gain (≥7% above baseline) occurred more commonly in the olanzapine group (20.2% vs. 1.9%; p = .001). By the end of the study, serum cholesterol (10.06 mg/dL vs. −0.96 mg/dL; p = .008) triglycerides (30.67 mg/dL vs. −4.17 mg/dL; p = .02), and LDL (8.78 mg/dL vs. -1.06 mg/dL, p = .01) had increased significantly more in the olanzapine group than in the placebo group. Compared to patients in the placebo group, serum HDL cholesterol had decreased significantly more in the olanzapine-treated group (-2.99 mg/dL vs. 1.10 mg/dL, p = .01).

Based on criteria established by the National Cholesterol Education Program for assessing lipid changes, a categorical change with potential clinical significance occurred with greater frequency in the olanzapine group than in the placebo group. Specifically, the percentage of patients who shifted from borderline to high fasting total cholesterol was significantly greater in the olanzapine group (0% vs. 25%, p = .007) (Table 5). Using the American Diabetes Association criteria for assessment of abnormal blood glucose levels, no significant differences between treatment groups were observed for categorical changes in blood glucose levels (Table 5).

**Table 5:** Absolute and categorical changes in weight, total cholesterol, triglycerides, LDL, HDL, and glucose

	**Placebo**		**Olanzapine**		**p Value**
	**n**	**LS mean (SE)**	**n**	**LS mean (SE)**	
Weight gain, kg	52	−0.36 (0.29)	104	+2.12 (0.21)	<.001
		**n/N (%)**		**n/N (%)**	
Patients with significant weight gain (≥7% body weight)		1/52 (1.9)		21/104 (20.2)	.001
	**n**	**LS mean (SE)**	**n**	**LS mean (SE)**	
Baseline-to-endpoint change in fasting laboratory values					
Cholesterol, mg/dL	52	−0.96 (3.37)	104	+10.06 (2.38)	.008
Triglycerides, mg/dL	52	−4.17 (12.31)	104	+30.67 (8.68)	.02
LDL, mg/dL	51	−1.06 (3.07)	103	+8.78 (2.16)	.01
HDL, mg/dL	52	+1.10 (1.27)	104	−2.99 (0.90)	.01
Glucose, mg/dL	51	−1.02 (1.11)	100	+ 1.01 (0.79)	.14
		**n/N (%)**		**n/N (%)**	
Patients with categorical shifts in fasting cholesterol values					
Normal to borderline		2/17 (11.8)		13/39 (33.3)	.11
Normal to high		0/17 (0.0)		1/39 (2.6)	1.00
Borderline to high		0/25 (0.0)		13/52 (25.0)	.007
Patients with categorical shifts in fasting triglyceride values					
Normal to borderline		3/37 (8.1)		5/82 (6.1)	.70
Normal to high		1/37 (2.7)		9/82 (11.0)	.17
Normal to extremely high		0/37 (0.0)		0/82 (0.0)	--
Borderline to high		1/9 (11.1)		3/12 (25.0)	.60
Borderline to extremely high		0/9 (0.0)		0/12 (0.0)	--
Patients with categorical shifts in fasting LDL cholesterol values					
Normal to borderline		4/9 (44.4)		11/26 (42.3)	>.99
Normal to high		0/9 (0.0)		0/26 (0.0)	--
Borderline to high		0/34 (0.0)		6/68 (8.8)	.18
Patients with categorical shifts in fasting HDL cholesterol values					
Normal to low		0/46 (0.0)		8/97 (8.2)	.05
Patients with categorical shifts in fasting glucose values					
Normal to impaired		5/32 (15.6)		9/72 (12.5)	.76
Impaired to high		0/18 (0.0)		2/28 (7.1)	.51
Normal/impaired to high		0/50 (0.0)		3/100 (3.0)	.55
Normal to high		0/32 (0.0)		1/72 (1.4)	1.00

Abbreviations: HDL = high-density lipoprotein; LDL = low-density lipoprotein; LS mean = least squares mean; SE = standard error.

There were no significant differences between groups in the proportion of patients with potentially clinically significant changes in vital signs and no clinically significant treatment-emergent electrocardiographic changes.

Compared with patients allocated to placebo, those allocated to olanzapine had small but statistically greater baseline-to-endpoint decreases in hemoglobin (-0.17 [0.42] g/L vs. −0.01 [SD 0.45] g/L; p = .04), total bilirubin (−1.07 [3.80] μmol/L vs. 0.71 [4.10] μmol/L; p = .02), and direct bilirubin (−0.33 [0.83] μmol/L vs. 0.15 [0.85] μmol/L; p = .001). Significant baseline-to-endpoint increases in gamma-glutamyltransferase (1.66 [28.8] μkat/L vs. −2.08 [12.9] μkat/L; p = .001) and prolactin (11.15 [19.69] pmol/L vs. −4.00 [25.14] pmol/L; p < .001) were noted for the olanzapine group compared to placebo. Baseline-to-endpoint change in hemoglobin A_1c_ did not differ between groups.

An assessment of suicidality (MINI Section C) revealed no significant difference between groups. The maximum increase from baseline in DIEPSS score was 0.33 (SD 0.76) in the olanzapine group (p < .001) and 0.19 (SD 0.56) in the placebo group (p = .03), with no significant difference between groups (p = .37).

## Discussion

The results seen with olanzapine monotherapy in Japanese patients with bipolar depression were consistent with those seen in the global parent study from which data for this subpopulation analysis were taken [14], and also with results from a prior study in which olanzapine monotherapy proved superior to placebo in reducing depressive symptoms in non-Asian patients with bipolar disorder experiencing an acute depressive episode [24]. Improvements in depressive symptoms were noted using multiple assessment tools (MADRS, HAMD-17, and CGI-BP).

Greater efficacy with olanzapine than with placebo was noted on individual items included in the MADRS. Specifically, patients in the olanzapine group reported less inner tension, less sleep reduction, better appetite, and less pessimism. Treatment effects appeared especially strong for the Reduced Sleep and Reduced Appetite MADRS items, and these findings may have clinical impact related to both safety and efficacy. Improvement of core symptoms of depression as assessed by the MADRS-6 subscale was significantly greater during treatment with olanzapine compared with placebo when analyzed with LOCF and MMRM methodologies, suggesting that olanzapine has an effect on the core symptoms of depression.

Although statistically significant differences in outcome measures were found between olanzapine and placebo, the differences were numerically small. In addition, the effect size demonstrated by olanzapine monotherapy in this population was modest. Although this could be indicative of a modest effect of the active drug, it may also have been influenced by a relatively strong placebo response. Increased placebo response is a phenomenon which has been noted in different areas of psychopharmacology in recent years [25,26]. Also, it is important to appreciate that bipolar disorder is particularly difficult to manage, and very few treatments have regulatory approval for treatment of bipolar depression. Therefore, even a modest effect size may be of clinical relevance to patients, although treatment-emergent adverse events need to be taken into consideration as well.

There were no significant treatment effects for rates of response, recovery, or remission. However, these rates represented dichotomous outcomes which, in some cases, were quite severe in their definition. This may have rendered possible differences over 6 weeks between groups difficult to detect. Also, these assessments may have been affected by the relatively small sample size of the subpopulation included in this analysis. It is interesting to note that the remission criterion defined a priori (MADRS total score ≤12) yielded a very high rate of remission in the placebo arm. A stricter criterion like the one recommended by ISBD (MADRS total score ≤8) may be preferable for signal detection in placebo-controlled clinical trials.

Japanese patients treated with olanzapine monotherapy in this study experienced no increase in manic symptoms; rather, mean total scores on the YMRS actually improved compared with those for patients treated with placebo. This result suggests that Japanese patients with bipolar depression who are experiencing a depressive episode may be able to be treated with olanzapine without increasing the risk of treatment-emergent mania. More importantly, this result suggests that symptoms of the opposite pole (mixed), a feature which is common in bipolar depression, improve with the use of olanzapine. The clinical importance of this finding was highlighted by the results of a recent study in which patients with bipolar disorder who exhibited more manic and hypomanic symptoms had less response to treatment than patients who had fewer of these symptoms [27].

The safety profile of olanzapine in this 6-week trial was similar to that seen in the parent study [14] from which these data were taken, and which had a more ethnically diverse patient population. Likewise, changes in weight and lipids were consistent with the known safety profile of olanzapine [28]. We noted greater weight gain in patients with bipolar depression in this population (+2.12 kg) than the weight gain observed in an earlier 6-week study of olanzapine for Japanese patients with bipolar mania [27] (+1.22 kg).

The primary limitation of this analysis of data from a Japanese subset of patients from a larger study was that it was an exploratory analysis with no adjustment for multiplicity. The study had fewer patients from which to draw conclusions to address questions of interest. For example, sample size may have been too small to reveal significant differences for dichotomous outcomes such as response and remission. Finally, the results presented here are limited to Japanese patients, age 18 to 64 years.

## Conclusions

Results of this analysis support the efficacy and tolerability of olanzapine in the treatment of Japanese patients with bipolar depression. Results in the Japanese population were consistent with the more ethnically diverse parent study. In making treatment decisions for individual patients, clinicians should carefully consider the risks and benefits of olanzapine treatment.

### Ethics approvals

Ethics committees at the following sites provided ethical approval for the study:

Institutional Review Board Yamaguchi University Hospital; Aino Clinic Institutional Review Board; Suzuki Internal & Circulatory Medical Clinic IRB; Himorogi Psychiatric Institute Institutional Review Board; Medical Corporation Cattleyakai Dr. Mano Medical Clinic Institutional Review Board; Kawaguchi Clinic Institutional Review Board; Medical Corporation Seikokai New Medical Research System Clinic Institutional Review Board; Institutional Review Board of CNS Yakurikenkyukai; Seimou Hospital Institutional Review Board; The Institutional Review Board, the University of Tokyo Hospital; Institutional review board of Nippon Medical School Hospital; Aichi Medical Association Institutional Review Board; Institutional Review Board of Fujita Health University Hospital; Institutional Review Board, Nippon Medical School Chiba Hokusoh Hospital; Mitsui Memorial Hospital Institutional Review Board; Japanese Red Cross Medical Center Institutional Review Board; and the Institutional Review Board of Ome Municipal General Hospital.

## Abbreviations

ANOVA: Analysis of variance; CGI-BP: Clinical Global Impressions-Bipolar Version Severity of Illness Scale; DIEPSS: Drug-induced Extrapyramidal Symptoms Scale; HAMD-17: 17-item Hamilton Depression Rating Scale; ISBD: International Society of Bipolar Disorder; LOCF: Last observation carried forward; MADRS: Montgomery-Åsberg Depression Rating Scale; MINI: Mini International Neuropsychiatric Interview; MMRM: Mixed-effects model repeated measures; SD: Standard deviation; SE: Standard error; YMRS: Young Mania Rating Scale.

## Competing interests

This study was funded by Eli Lilly and Company. Drs. McDonnell, Katagiri, and Gomez, and Mr. Case and Mr. Fujikoshi are full-time employees and minor stockholders of Eli Lilly and Company. Dr. Takahashi was a contract employee working for Eli Lilly Japan and a minor stockholder in Eli Lilly and Company. Dr. Tohen was an employee of Eli Lilly and Company while the study was conducted (up to 2008) and has received honoraria from or consulted for AstraZeneca, Bristol Myers Squibb, Glaxo-SmithKline, Eli Lilly and Company, Johnson & Johnson, Sepracor, Otsuka, Merck, Sunovion, Forest, Lundbeck, Wyeth, and Wiley Publishing; his spouse is a current employee and minor stockholder at Eli Lilly and Company. Dr. Kanba received grants and research support from GlaxoSmithKline, Pfizer, Yoshitomi, Kyowa-Hakko, Astellas, Meiji, and Otsuka; was a consultant for Eli Lilly and Company, GlaxoSmithKline, Pfizer, Ono, Asahi-kasei, Shionogi, and Otsuka; and received honoraria from Eli Lilly and Company, GlaxoSmithKline, Pfizer, Janssen, Meiji, Kyowa-Hakko, Dainippon-Sumitomo, Otsuka, Eisai, Taisho-Toyama, and Astellas.

## Authors’ contributions

Dr. Katagiri contributed to the acquisition of data, analysis and interpretation of data, drafting portions of the manuscript, and critically revising manuscript for important intellectual content. Dr. Tohen contributed to the conception and design of the study, acquisition, analysis, and interpretation of data; drafted portions of the manuscript, and critically reviewed the manuscript. Drs. McDonnell, Kanba, Takahashi, and Gomez and Mr. Fujikoshi and Mr. Case contributed to the analysis and interpretation of data; drafted portions of the manuscript, and critically reviewed the manuscript. All authors read and approved the final manuscript.

## Pre-publication history

The pre-publication history for this paper can be accessed here:

http://www.biomedcentral.com/1471-244X/13/138/prepub

## Supplementary Material

Additional file 1: Figure S1 Visit-wise change from baseline in LS mean MADRS total score (Observed cases methodology). Abbreviations: LS = least squares; MADRS = Montgomery-Åsberg Depression Rating Scale; OC = observed case. Click here for file

Additional file 2: Figure S2 Visit-wise change from baseline in LS mean MADRS total score (Mixed-effects model repeated measures methodology). Abbreviations: LS = least squares; MADRS = Montgomery-Åsberg Depression Rating Scale; MMRM = mixed-effects model repeated measures. Click here for file
