# Supplementary figures and images for: Efficacy and safety of olanzapine for treatment of patients with bipolar depression: Japanese subpopulation analysis of a randomized, double-blind, placebo-controlled study
Source: BMC Psychiatry. 2013 May 14;13:138. doi: 10.1186/1471-244X-13-138 (PMC3666902; doi:10.1186/1471-244X-13-138)

**Supplemental Figure 1.**


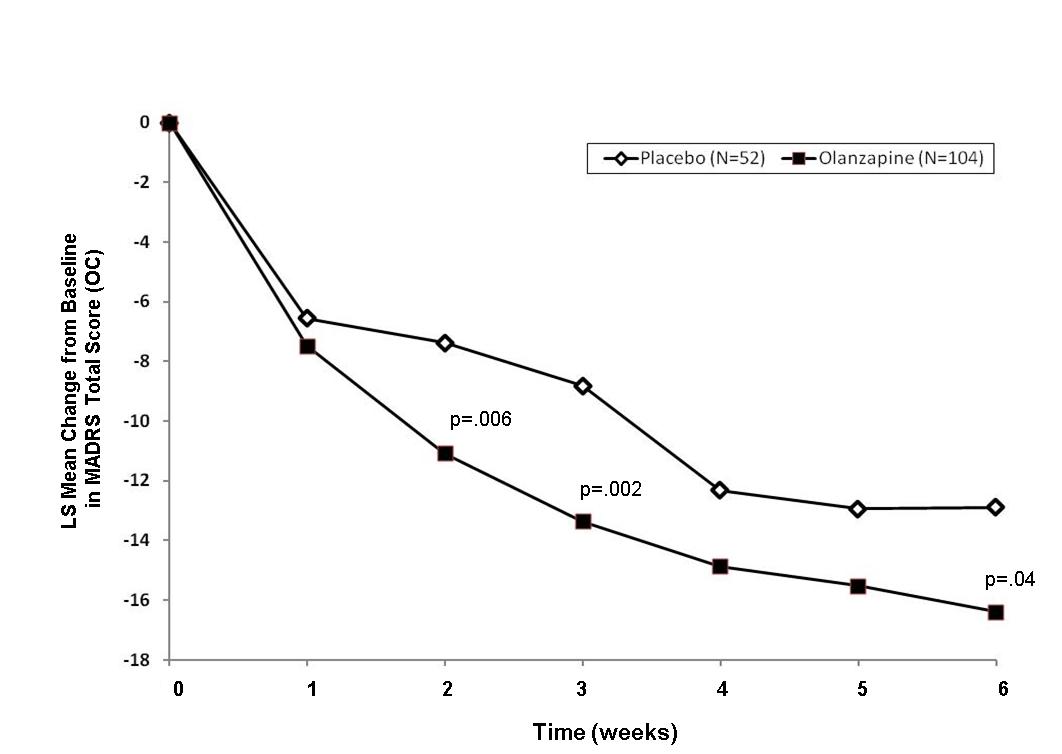

Supplement: Additional file 1: Figure S1 — Visit-wise change from baseline in LS mean MADRS total score (Observed cases methodology). Abbreviations: LS = least squares; MADRS = Montgomery-Åsberg Depression Rating Scale; OC = observed case. [file 1471-244X-13-138-S1.docx]

**Supplemental Figure 2.**


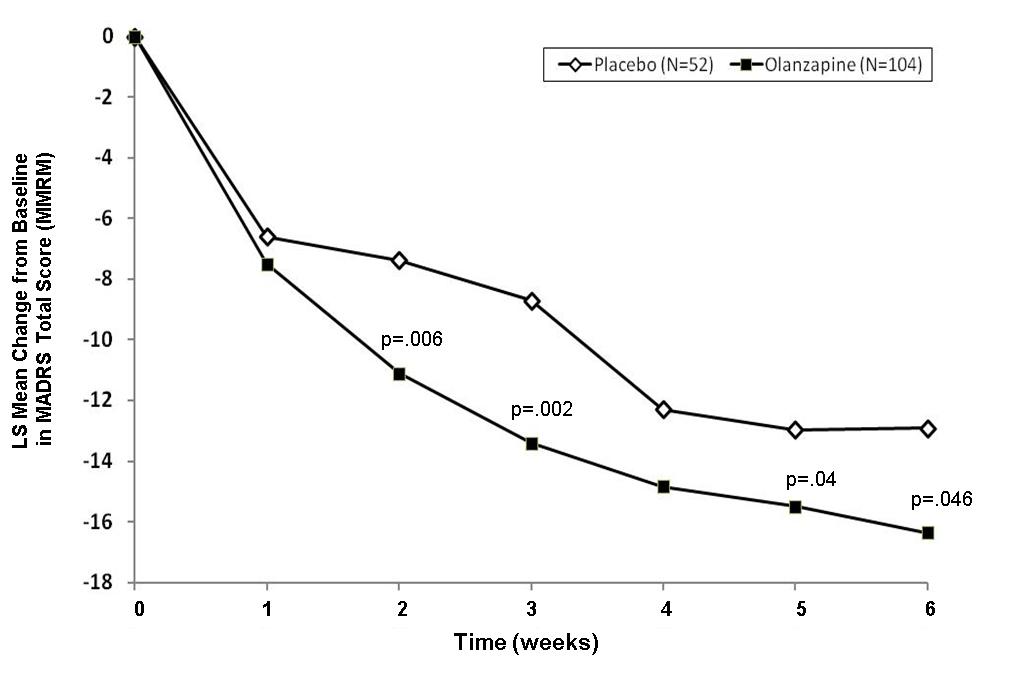

Supplement: Additional file 2: Figure S2 — Visit-wise change from baseline in LS mean MADRS total score (Mixed-effects model repeated measures methodology). Abbreviations: LS = least squares; MADRS = Montgomery-Åsberg Depression Rating Scale; MMRM = mixed-effects model repeated measures. [file 1471-244X-13-138-S2.docx]
